# Ostracods had colonized estuaries by the late Silurian

**DOI:** 10.1098/rsbl.2021.0403

**Published:** 2021-12-01

**Authors:** Anna McGairy, Toshifumi Komatsu, Mark Williams, Thomas H. P. Harvey, C. Giles Miller, Phong Duc Nguyen, Julien Legrand, Toshihiro Yamada, David J. Siveter, Harrison Bush, Christopher P. Stocker

**Affiliations:** ^1^ School of Geography, Geology and the Environment, University of Leicester, Leicester LE1 7RH, UK; ^2^ Faculty of Advanced Science and Technology, Kumamoto University, Kumamoto, 860-8555, Japan; ^3^ The Natural History Museum, Cromwell Road, South Kensington, London SW7 5BD, UK; ^4^ Department of Paleontology and Stratigraphy, Vietnam Institute of Geosciences and Mineral Resources, 100000 Hanoi, Vietnam; ^5^ Department of Geosciences, Faculty of Science, Shizuoka University, Shizuoka, 422-8529, Japan; ^6^ Botanical Gardens, Faculty of Science, Osaka City University, Osaka, 576-0004, Japan

**Keywords:** Silurian, ostracods, pioneer, colonizers, estuary

## Abstract

The fossil record of terrestrialization documents notable shifts in the environmental and physiological tolerances of many animal and plant groups. However, for certain significant components of modern freshwater and terrestrial environments, the transition out of marine settings remains largely unconstrained. Ostracod crustaceans occupy an exceptional range of modern aquatic environments and are invaluable palaeoenvironmental indicators in the fossil record. However, pre-Carboniferous records of supposed non-marine and marginal marine ostracods are sparse, and the timing of their marine to non-marine transition has proven elusive. Here, we reassess the early environmental history of ostracods in light of new assemblages from the late Silurian of Vietnam. Two, low diversity but distinct ostracod assemblages are associated with estuarine deposits. This occurrence is consistent with previous incidental reports of ostracods occupying marginal and brackish settings through the late Silurian and Devonian. Therefore, ostracods were pioneering the occupation of marginal marine and estuarine settings 60 Myr before the Carboniferous and they were a component of the early phase of transition from marine to non-marine environments.

## 1. Introduction

Identifying the physiological adaptations and environmental contexts that allowed marine organisms to colonize brackish and freshwater niches is necessary to understand the development of complex terrestrial ecologies. Ostracods are the most abundant arthropods in the fossil record and occupy basal positions in aquatic trophic networks [1]. Despite their abundance and ubiquity as primary consumers and detritivores in aquatic settings today, and their widespread use as palaeoenvironmental indicators in the fossil record, the timing of their transition from marine to non-marine aquatic niches remains unclear. With fossil evidence of complex terrestrial ecosystems by the late Silurian [2–4], including both early vascular plants and predatory arthropods, the apparent absence of contemporaneous ostracods in non-marine aquatic settings presents a conundrum.

The fossil record of marine ostracods extends to the earliest Ordovician [5], and possibly the Cambrian [6]. There are brief descriptions of ostracods in marginal marine or brackish-water settings from the late Silurian and Devonian [7–12], but currently, the earliest accepted occurrence of ostracods in non-marine aquatic settings, supported by extensive sedimentological evidence, is that of the early Carboniferous at *ca* 350 Ma [13–15].

Here, we document faunas from the late Silurian, *ca* 423 Ma, Si Ka Formation of northern Vietnam (electronic supplementary material, figure S1) that points to an early colonization of estuarine settings by ostracods. We explore the reasons for this colonization and its significance for the development of non-marine aquatic ecologies.

## 2. Material and methods

**Figure 1.**
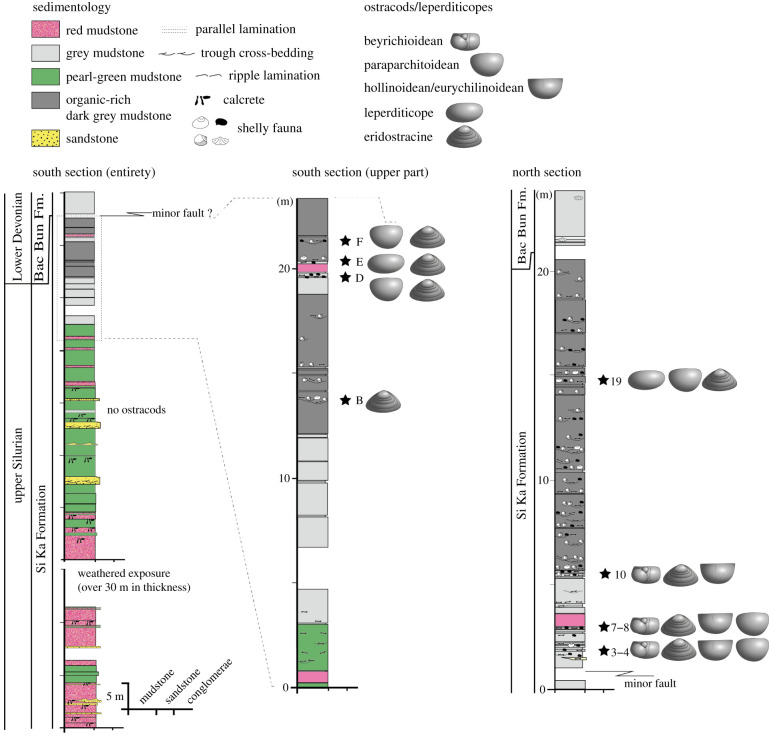
Ostracod assemblages in the late Silurian Si Ka Formation, road section between Lung Cu and Ma Le, Dong Van District, northern Vietnam (for location see electronic supplementary material, figure S1). Ostracods are absent from the fluvial and floodplain (coloured red and green) deposits of the lower and middle Si Ka Formation, but two assemblages are identifiable in the upper (grey) estuarine deposits. Assemblage 1, horizons 3–4, 7–8 and 10, is characterized by hollinoideans, eurychilinoideans, beyrichioideans, eridostracines and paraparchitoideans; Assemblage 2, horizons 19, B, D–F, is characterized by paraparchitoideans and eridostracines.

**Figure 2.**
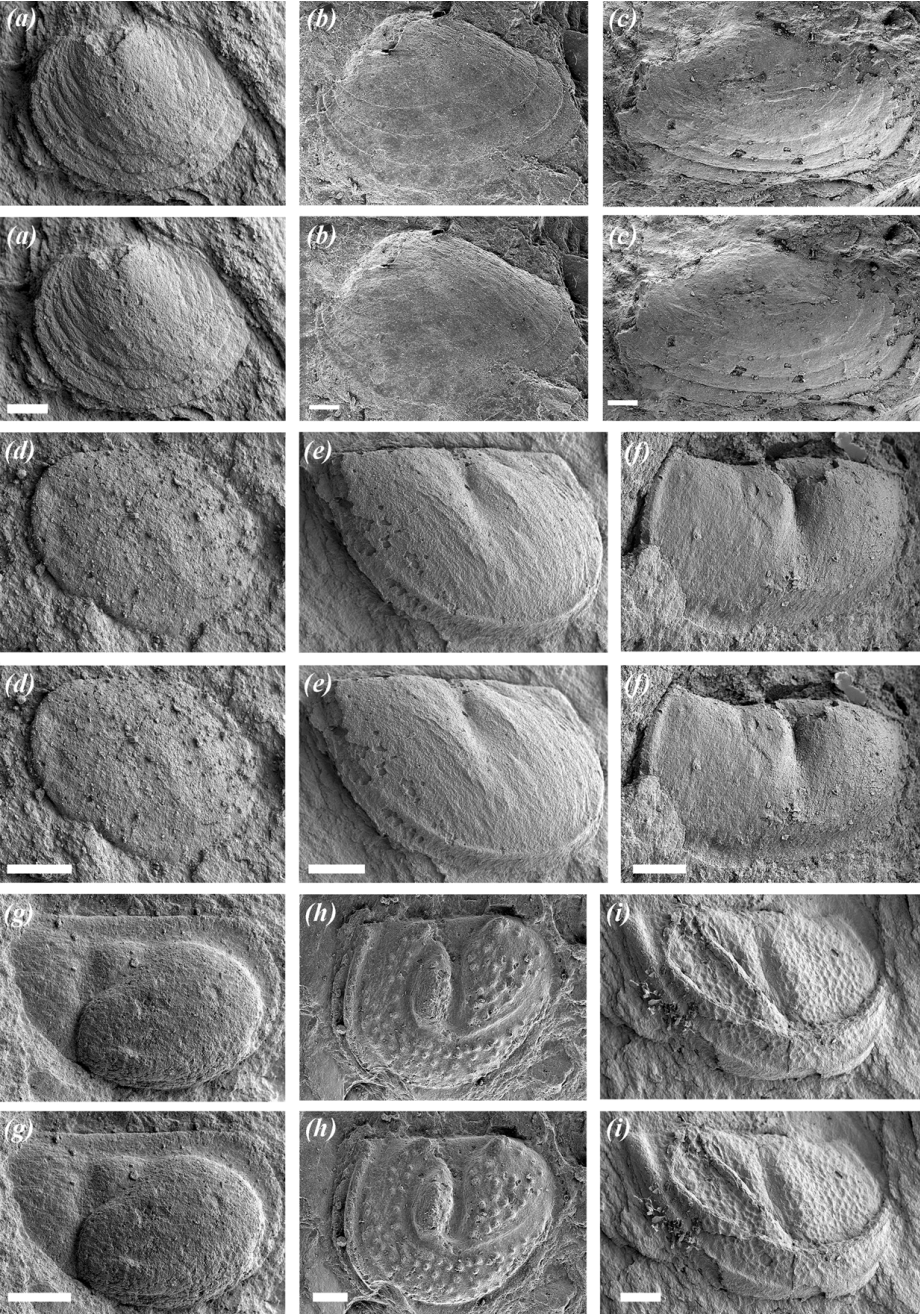
Scanning electron photomicrographs (stereo-pairs) of silicone rubber casts of ostracods from the Si Ka Formation. Repository numbers are DGMV. Assemblage 1 includes all taxa figured here. Assemblage 2 is typified by the eridostracines and paraparchitoidean. (*a*) eridostracine sp. 1, BT5/531a; (*b*) eridostracine sp. 3, BT9/531a; (*c*) eridostracine sp. 2, BT8/531a; (*d*) paraparchitoidean sp., BT21/531a; (*e*) hollinoidean sp. 1, BT3/531a; (*f*) eurychilinoidean sp., BT15/531a; (*g*) beyrichioidean sp. 1, BT11/531a; (*h*) beyrichioidean sp. 2, BT18/531a; (*i*) hollinoidean sp. 2, BT3/531b. All scale bars: 250 µm.

Ostracod-bearing mudstones from eight horizons in the upper part of the Si Ka Formation have been examined (figure 1), together with loose material collected adjacent to outcrop, but unlocalized. More than 120 rock slabs were studied, and these preserve several hundred ostracod specimens as external and internal moulds. Fossils were cast using the silicone rubber ‘Silcoset 101' [16]. Specimens were first consolidated using a solution of 1% ‘Paraloid B-72' (https://www.zoicpalaeotech.co.uk/) in acetone. To prevent the silicone from adhering to the specimen ‘Ambersil HD' silicone release agent was applied to the consolidated surface prior to casting. Over 160 ostracod casts have been imaged using a Hitachi S-3600N environmental scanning electron microscope (figure 2). All rock slabs, ostracod fossils and casts are stored at the Geological Museum, General Department of Geology and Minerals of Vietnam (DGMV), Hanoi: figured specimens are on rock slabs numbered BT1 to BT22. To establish depositional environment, sections were logged for sedimentology, and a facies analysis based on the lithology, sedimentary structures, grain size and fossils of the deposits was undertaken through the Si Ka Formation (figure 1; electronic supplementary material, figure S2).

## 3. Results

Palynological data indicate the Si Ka Formation is of Silurian, late Ludlow to early Pridoli age [17]. It unconformably overlies the lower Palaeozoic Lutxia and Than Sa formations, and is succeeded by the shelf marine-deposited mudstones of the Lower Devonian Bac Bun Formation [18–20]. The Si Ka Formation formed in a subtropical setting on the South China palaeo-plate [21]. The lower and middle part of the Si Ka Formation consists mainly of red, pearl green and grey mudstones and sandstones (figure 1, electronic supplementary material, figure S2). The sandstones (0.5–2 m thick) commonly contain trough cross-stratification and parallel lamination, and typically represent the base of fining-upwards sequences characterized by erosional surfaces and rip-up clasts, overlain by vari-coloured mudstones (1–8 m thick). Some of these mudstones are red in colour and contain tubular, spheroidal and lenticular calcretes [22].

The upper 25 m of the Si Ka Formation is dominated by organic-rich, dark grey mudstones intercalated with silty mudstone layers and shell and/or bone beds and it is these units that contain ostracods (figure 1; electronic supplementary material, figure S2). The mudstones often yield thin ‘coal' layers, plant debris and plant spores [17]. Fragments of antiarch fish bones (e.g. *Yunnanolepis*), lingulid brachiopods, gastropods, bivalves and ichnofossils are present. The fossils comprise low diversity and moderately high density assemblages. Typical stenohaline marine taxa, such as echinoderms, cephalopods, trilobites and corals are absent.

Two ostracod assemblages are differentiated: (i) a higher abundance but low diversity assemblage of ten species including beyrichioideans and (ii) a low abundance and low diversity assemblage of eridostracines and paraparchitoideans co-occurring with leperditicope arthropods (some authors include the latter in the Ostracoda, see [23]), but lacking beyrichioideans (figure 1).

*Assemblage 1* (figure 1, horizons 3–4, 7–8 and 10) is associated with grey mudstone and sandy mudstone, interpreted as estuarine facies [22]. It comprises three eridostracine species, two beyrichioidean species, two hollinoidean species, a eurychilinoidean, a paraparchitoidean (figure 2) and an indeterminate palaeocopid; for morphology of lower Palaeozoic ostracods see [24]. It typically occurs in thin (centimetric-scale) shell lags associated with fragments of plant debris, fish and bivalve molluscs (including Pterineidae), and a microflora of trilete spores. These horizons do not include leperditicopes. It includes both juvenile and adult ostracods, with many valves intact. Horizons 3–4 and 7–8 (figure 1) immediately overlie basal lag deposits (1–8 cm thick) characterized by erosional basal surfaces, composed mainly of fragments of gastropods, pterineid bivalves and rip-up clasts.

*Assemblage 2* (figure 1, horizons 19, B, D–F) is associated with grey mudstone and sandy mudstone lithologies, also interpreted as estuarine facies. It comprises the eridostracines and paraparchitoidean of Assemblage 1, and rare, singular occurrences of indeterminate palaeocopids. The ostracods are found either scattered across the slab or in shell lags alongside fragments of gastropods, fish, leperditicopes and bivalves (electronic supplementary material, figure S3). The abundance of ostracods relative to other fauna is low, and there is little to no associated macro-plant debris, although trilete spores are present [17]. Both juvenile and adult ostracods are present.

In neither of the two ostracod assemblages is there a size bias or evidence of a preferred valve alignment or stacked valves, as determined from the preservation of the ostracods on rock slabs. Some moulds indicate that carapaces were preserved articulated with the valves in ‘butterfly’ orientation, suggesting minimal agitation. Adults and juveniles of individual species often co-occur. Many valves were evidently preserved complete, and although some are fractured, in many cases this appears to be post-deposition. Horizons with shell lags suggest local transport, but overall, both ostracod assemblages appear representative of life assemblages [25].

## 4. Discussion

Our data suggest that a systematic analysis of late Silurian and Devonian, and perhaps earlier [26], marginal marine and non-marine aquatic sedimentary deposits may identify ostracod pioneers in these settings, tens of millions of years earlier than previously supposed.

Sedimentological and palaeontological data indicate a fluvial–estuarine setting for the Si Ka Formation [22]. The associated micro- and macrofossils include a notable absence of stenohaline marine indicators such as acritarchs or corals, and are typified by plant and fish debris, bivalves, gastropods, leperditicopes and trilete spores [17]. The fining-upward sandstones of the lower and middle parts of the Si Ka Formation (figure 1; electronic supplementary material, figure S2) represent typical fluvial channel-fill deposits, while the vari-coloured mudstones were probably deposited on a floodplain. The red mudstone likely indicates accumulation in an oxidized state in arid to semi-arid climates.

The fossiliferous grey mudstones from the upper 25 m of the formation (figure 1; electronic supplementary material, figure S2) might plausibly be interpreted as a low salinity sea, similar to the Baltic, or as coastal/estuarine settings. We favour interpretation as a central estuarine environment, indicated by levels with red beds (figure 1), but some lag deposits in shell and/or bone beds seem to be transported from the outer to central estuary, perhaps in marine flooding or storm events. Although the palaeoecology and habitat of almost all molluscs from the Si Ka Formation are unknown, pterineid bivalves are typically of marine origin [27]. Some species of the latest Silurian to Early Devonian antiarch fish were probably transitioning from marine to non-marine aquatic environments during this time [28]. Lingulid brachiopods suggest a marginal marine influence, as might be expected in an estuary.

The Si Ka Formation hosts Silurian ostracod taxa that are conventionally considered as marine [29,30], especially beyrichioideans, eridostracines, eurychilinoideans and hollinoideans of Assemblage 1 (figure 2). However, beyrichioideans are known from very marginal marine facies of the late Wenlock, *ca* 428 Ma, Straiton Grits Formation in Scotland [10,31], the late Silurian Downton Castle Sandstone Formation in the Welsh Borderland, *ca* 423 Ma [8,32], from the Early Devonian Khao Loc Formation of Vietnam [11] and from Middle Devonian floodplain and estuarine deposits of the Catskill Mountains, New York State [9], suggesting that some were euryhaline. Additionally, eridostracines are known from littoral settings of the Devonian [33–35]. Therefore, we interpret Assemblage 1 as a marginal marine, brackish-tolerant assemblage, either living at the mouth of the Si Ka estuary, or influenced by marine flooding or storm events that transported in more marine taxa, such as beyrichioidean ostracods and pterineid bivalves, cf. [36]. This interpretation is supported by the associated sedimentology and palaeontology (electronic supplementary material, figure S2) and by the overall low diversity of the assemblage, which is atypical of fully marine Silurian ostracod assemblages [29,30,37] but is typical of stressed or brackish-water ostracod assemblages [38].

Assemblage 2 contains paraparchitoideans, eridostracines, leperditicopes and occasional indeterminate palaeocopids. Leperditicopes are known from marginal marine settings as early as the Ordovician [39], suggesting an ability to tolerate a wide range of salinities. Paraparchitoideans are recorded from marine environments of the late Silurian [40], and the group diversified in the Devonian and Carboniferous. Low diversity assemblages of paraparchitoideans are typical of settings with brackish and fluctuating salinities in the Carboniferous [33,36,41,42]. Based on the distribution of taxa, their sedimentological setting and the co-floral and faunal associations, we interpret this assemblage to be adapted for an estuary.

Modern brackish-water and estuarine ostracod assemblages are typically high abundance and low diversity, as expected for a stressed environment with rapidly fluctuating salinity conditions [38,43,44]. In tropical estuaries, salinity fluctuates annually with monsoonal precipitation, ranging from essentially freshwater to marine salinities [45]. In such settings ostracod abundance varies accordingly and is lowest during the monsoon season [45,46].

Marine, brackish and freshwater species can co-occur in modern estuaries if their salinity tolerances overlap [38,47–49]. Modern brackish-water environments host a combination of truly brackish-water ostracods alongside non-marine taxa that are tolerant of raised salinities and/or marine taxa that are tolerant of reduced salinities [47]. The overall reduced diversity in brackish settings allows true brackish-water taxa to achieve high population densities due to the lack of competition. The combination of ostracod taxa in an assemblage can provide information on salinity [38].

The wide environmental distribution of extant and fossil ostracods suggests that the physiological tolerance of a range of salinities may be deep-rooted in the group [50], and this may have favoured multiple attempts at colonizing estuaries and non-marine aquatic settings: it is notable that none of the taxa we describe here are lineages traceable into modern non-marine ostracods. To be successful in colonizing non-marine aqueous environments, ostracods would have required the ability to regulate their internal salinity [14]. The lack of ornamentation and soft-part preservation in the ostracods of the Si Ka Formation provides no visual aids to interpret how they managed osmoregulation. However, the presence of two distinct ostracod assemblages, with some overlapping taxa (notably eridostracines and a paraparchitoidean), hints at salinity tolerance. Ostracods also adopt reproductive strategies that would be advantageous in stressed environments, notably brood care, parthenogenesis or desiccation-resistant eggs [13]. We have no evidence for the latter two strategies in the Si Ka Formation, but at least one each of the beyrichioidean and hollinoidean species are dimorphic, and the former are known to have brooded live young [51].

The colonization of freshwater environments may be either passive or active [52]. In active colonization, organisms exploit the benefits of a new environment, for example, a previously untapped food source and lack of competition. Estuaries provide conduits for active invasion that have been used by many organisms, notably fish and crustaceans, but also trilobites during the early Palaeozoic [53]. Alternatively, falling sea levels may lead to passive invasion by stranding organisms in water bodies that freshen over time. We suggest that ostracods were actively invading the Si Ka estuary, this transition being facilitated by physiological adaptation to salinity variation.

A broad review of estuarine trace fossils through time suggests five major phases of brackish-water colonization, including one from the Silurian through to the Carboniferous [54]. Bioturbated floodplains are recognizable from the late Silurian, coinciding with the rise in vascular land plants [55]. Reconstructions of late Silurian–Devonian aquatic and semi-aquatic terrestrial trophic structures are not dissimilar to those of modern ecosystems, though with the notable absence of terrestrial vertebrates [4,56]. Primary consumers were likely detritivores or deposit feeders, with carnivorous secondary consumers such as eurypterids or fish [4]. It has been suggested that the radiation of vascular land plants will have introduced vegetative detritus into fluvial systems and encouraged animals to disturb the sedimentary substrate foraging for nutrients [55,57]. Coprolites from the late Silurian Welsh Borderland provide evidence for the presence of detritivores in the Pridoli [58,59]. In the Si Ka estuary, ostracods would have occupied the position of primary consumers as detritivores, scavenging plant matter and in turn providing a food source to secondary consumers like fish or carnivorous arthropods. Furthermore, the development of meandering rivers aided by the rise of rooting land plants would have resulted in increased complexity and abundance of vegetation along channels [60,61], and an improved food supply for detritivores.

Our data from the Si Ka Formation show that ostracods were already pioneers of estuarine environments by the late Silurian. Their presence in such settings, at the interface between marine and non-marine environments, suggests that they were in the vanguard of colonizing early terrestrial ecosystems.

## Data accessibility

Additional materials to support this manuscript, including the location of the road sections, sedimentology and taphonomy, are included in electronic supplementary figures S1–S3. Details of museum numbers for ostracod specimens are given in electronic supplementary material, text. The data are provided in the electronic supplementary material [62].
